# Metastatic Breast Cancer in Kenya: Presentation, Pathologic Characteristics, and Patterns—Findings From a Tertiary Cancer Center

**DOI:** 10.1200/JGO.19.00036

**Published:** 2019-07-10

**Authors:** Etoroabasi Ekpe, Asim Jamal Shaikh, Jasmit Shah, Judith S. Jacobson, Shahin Sayed

**Affiliations:** ^1^ Columbia University Vagelos College of Physicians and Surgeons, New York, NY; ^2^ Columbia University Mailman School of Public Health, New York, NY; ^3^ Aga Khan University Hospital, Nairobi, Kenya

## Abstract

**PURPOSE:**

The purpose of this research was to describe the sociodemographic and clinical characteristics of Kenyan women with metastatic breast cancer diagnosed and treated at Aga Khan University Hospital in Nairobi, Kenya from 2012 to 2018.

**PATIENTS AND METHODS:**

We reviewed charts of Kenyan women with metastatic breast cancer and analyzed sociodemographic data, breast cancer risk factors, and tumor characteristics associated with stage at diagnosis, receptor status (ie, estrogen receptor, progesterone receptor, and human epidermal growth factor receptor 2 [HER2]), and site of metastasis using χ^2^, analysis of variance, two-sample *t* tests, and logistic regressions.

**RESULTS:**

A total of 125 cases with complete medical records were included in the analysis. Forty women (32%) had metastases at diagnosis. Of the others, those diagnosed in stage III developed metastases sooner than those diagnosed in stage II (*P* < .001). Fifty-eight percent of patients had metastases to bone, 14% to brain, 57% to lungs, and 50% to liver. Seventy-four percent of patients presented with more than one metastatic site. Metastases to bone were associated with greater age at diagnosis (*P* = .02) and higher parity (*P* = .04), and metastases to the brain were associated with early menopause (*P* = .04), lower parity (*P* = .04), and lack of breastfeeding (*P* = .01). Patients whose tumors were triple negative (estrogen receptor-negative, progesterone receptor-negative, and HER2 negative) were more likely to develop brain metastases (*P* = .01), and those whose tumors were HER2 positive were more likely to develop liver metastases (*P* = .04).

**CONCLUSION:**

Although our data on patterns of metastases and pathologic subtypes are similar to those in published literature, some unique findings concerning hormonal risk factors of women with metastatic breast cancer and specific metastatic sites need additional exploration in larger patient populations.

## INTRODUCTION

In Kenya, the age-standardized rate of breast cancer incidence is 40.3 per 100,000 women, making it the most common cancer among Kenyan women.^1^ In 2013, the reported mortality rate from breast cancer in Kenya was 7.1 per 100,000 women.^2^ Corresponding rates in the United States in 2015 are 124.8 and 20.3 per 100,000 women for incidence and mortality, respectively.^3^ In sub-Saharan Africa, the proportion of patients with breast cancer with late stage (III or IV) disease at diagnosis ranges from 30% in South Africa to 98% in Nigeria.^4^ In Kenya, 42% of women with breast cancer present in stage III and 18% in stage IV (metastatic disease).^5^ In the United States, only 5% to 8% of patients with breast cancer present with stage IV disease.^6^

Common sites for breast metastases are the lungs, liver, bones, and brain.^7^ The development of metastatic disease is often unpredictable but has been associated with the initial stage at diagnosis and with other tumor characteristics, including receptor status.^8^ All subtypes of breast cancer on the basis of hormonal and human epidermal growth factor receptor 2 (HER2) receptor status have the potential to become metastatic.^7^ Other risk factors for metastases include age, nodal status, tumor grade, and size.^9^

Few studies have focused on breast cancer characteristics in African populations; of those published, most have reported on the molecular profile and clinical staging of patients with breast cancer.^10-13^ High-income countries have documented metastatic disease patterns in relation to receptor status and molecular subtype of breast cancer.^8,14-16^ No studies from sub-Saharan Africa have specifically described patterns of metastatic breast cancer with patient and tumor characteristics.

The purpose of this study was to describe sociodemographic, clinical, and pathologic characteristics of a series of Kenyan women with metastatic breast cancer. We analyzed metastatic patterns in this patient population and identified factors associated with site of metastasis, stage at initial diagnosis, and receptor status.

## PATIENTS AND METHODS

### Patients

Patients with metastatic breast cancer from Kenya with complete medical records who were diagnosed and treated at Aga Khan University Hospital between January 2012 and August 2018 were included in this retrospective study. We analyzed sociodemographic data, breast cancer risk factors, and tumor characteristics associated with stage at diagnosis, receptor status (ie, estrogen receptor [ER], progesterone receptor [PR], and HER2), and site of metastasis. Treatment in the metastatic setting included chemotherapy with taxane- or nontaxane-based therapy; radiation at either the breast or metastatic site; hormonal therapy with tamoxifen, letrozole, or anastrozole; and trastuzumab-based therapy for those who were HER2 positive.

### Hospital Site

Aga Khan University Hospital (AKUH) in Nairobi, Kenya, is a 250-bed nonprofit institution with a well-established cancer center. Its Comprehensive Cancer Program provides care that meets the highest international standards of practice to patients with cancer throughout East Africa.^17^ The center provides screening, treatment, palliative care, and rehabilitation programs. Services include nuclear medicine, surgery, chemotherapy, radiotherapy, and pathology laboratories.

### Ethical Approval

This study was approved by AKUH’s institutional review board (Ref: 2018/REC-54 [v1]) and all data for analysis were collected and de-identified by an AKUH research nurse. Columbia University’s institutional review board exempted the study from review because study personnel worked with de-identified data only.

### Data Abstraction

AKUH study personnel abstracted all data from electronic medical records and pathology reports. The data set included clinical and demographic information, dates of diagnosis of primary and metastatic disease, stage at diagnosis, site of metastases, and histology of primary tumor and metastases (including molecular phenotype of primary tumor on the basis of ER, PR, and HER2 expression).

### Sociodemographic and Reproductive Risk Factors

Sociodemographic and reproductive risk factors from chart review included age at initial breast cancer diagnosis, age at menarche, age at menopause, menopausal status, parity or number of childbirths, body mass index, breastfeeding status, contraception use, family history of cancer, educational level, smoking history, and alcohol use. All data including age, body mass index, and menopausal status were based on the time at original breast cancer diagnosis. Contraception use was defined as any use of hormonal contraception, including oral contraceptive pills and long-acting reversible contraceptives. Family history of cancer was defined as any first-degree relative diagnosed with any form of cancer. Educational level was defined by completion of any accredited primary, secondary, or tertiary school. Smoking or alcohol use was defined as any patient-reported use of tobacco cigarettes or standard alcohol drinks.

### Histopathology and Immunohistochemistry

Details of histopathology and immunohistochemistry procedures at AKUH have been previously described.^5^ All biomarker testing was performed at the AKUH pathology laboratory, which is a College of American Pathologists–accredited laboratory. Histopathology and prognostic parameters included stage at diagnosis, tumor size, tumor type, grade, lymph node positivity, and ER/PR/HER2 status. Hormone receptor status was interpreted using the Allred scoring system, and HER2 status was interpreted using the ASCO/College of American Pathologists scoring system.^18,19^ Molecular subtypes were based on receptor status alone and organized in the following categories: (1) ER positive and/or PR positive, and HER2 negative; (2) ER positive and HER2 positive; (3) ER negative and HER2 positive; and (4) ER negative, PR negative, and HER2 negative (triple negative).

### Metastatic Site

Metastases were categorized as bone, brain, lung, and liver. Patients were evaluated for metastases by x-rays (lungs/pleura and bone), ultrasound (liver), bone scan (bone), magnetic resonance imaging (brain), and computed tomography (lung, liver, and brain).

### Statistical Analysis

Data were exported to and analyzed using Statistical Analysis Software (SAS 9.4; SAS Institute, Cary, NC). Descriptive statistics were presented as frequencies and percentages for categorical variables and as means and standard deviations for continuous variables. Categorical variables were analyzed using χ^2^ or Fisher’s exact tests, and continuous variables were analyzed using *t* test or analysis of variance tests to assess the relationships of sociodemographic risk factors and tumor characteristics with stage at diagnosis, pathologic subtype, and site of metastasis. Odds ratios (ORs) were calculated using logistic regressions for significant associations where appropriate.

## RESULTS

A total of 1,074 breast cancer cases were diagnosed and treated at Aga Khan University Hospital during the study period. One hundred thirty-seven were diagnosed with metastatic breast cancer, and 125 metastatic breast cancer cases were ultimately included in the study. Those excluded (n = 12) did not have complete follow-up data. All patients included in the study were black African women between the ages of 21 and 79 years. Their mean age at diagnosis was 47 ± 12 years, and their mean age at menarche was 13.1 ± 1.5 years. Among the 54 postmenopausal patients (43%), the mean age at menopause was 49.2 ± 1.5 years. Eleven patients (19%) were nulliparous, and the other 114 averaged three children per woman; all but one of the mothers breastfed. Sixty-seven percent of the patients reported ever having used some form of contraception, and 23% had a family history of cancer, eight of which were specific for breast cancer. The majority of patients (99%) had some form of schooling, and 50% had attended university. Only two patients (1.6%) had a history of smoking cigarettes, and 19 patients (15%) patients had ever used alcohol (Table 1).

**TABLE 1:**
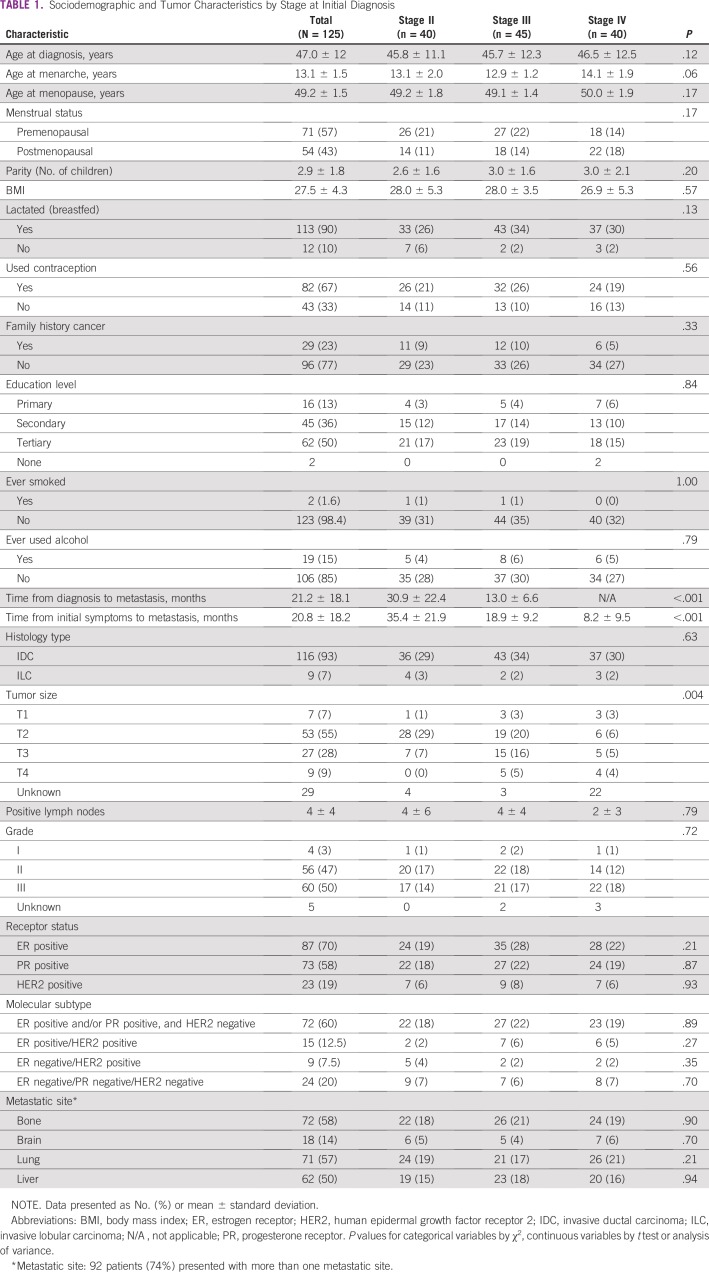
Sociodemographic and Tumor Characteristics by Stage at Initial Diagnosis

No patients were initially diagnosed at stage I; 40 patients (32%) were diagnosed at stage II, 45 (36%) at stage III, and 40 (32%) at stage IV (de novo metastases). The majority of patients had invasive ductal carcinoma (93%), with tumor grade of 2 (47%) or 3 (50%). Most tumors were ER positive (70%) and PR positive (58%); 19% of patients had HER2-positive breast cancer. Sixty percent of patients had ER-positive and/or PR-positive, HER2-negative molecular subtype; 12.5% of patients had ER-positive/HER2-positive; 7.5% had ER-negative/HER2-positive; and 20% had triple-negative tumors. Fifty-eight percent of patients had metastases to bone, 14% to brain, 57% to lungs, and 50% to liver. Ninety-two patients (74%) developed metastases at more than one site (Table 1).

### Breast Cancer Stage at Diagnosis

A significant association was found between later stage at diagnosis and shorter duration of time to metastatic disease: patients diagnosed in stage III developed metastases sooner than those diagnosed in stage II (OR, 0.89; *P* < .001), averaging at 13.0 months at stage III compared with 30.9 months at stage II. The time between initial symptoms of breast cancer and metastatic disease was also found to be significantly associated with stage (OR, 0.88; *P* < .001); the average time between initial symptoms of breast cancer and diagnosis of metastatic disease was 35.4 months for patients with stage II disease, 18.9 months for stage III, and 8.2 months for stage IV (de novo metastasis). Tumor size was also associated with stage at initial diagnosis (*P* = .004; Table 1).

### Breast Cancer Receptor Status/Molecular Subtype

Significant associations were found between PR-positive status and contraceptive use (OR, 2.83; *P* = .01) and between HER2-positive status (irrespective of ER/PR status) and younger age at menarche (OR, 0.65; *P* = .02). Receptor status did not seem to be associated with either stage at diagnosis or tumor grade (Table 2).

**TABLE 2:**
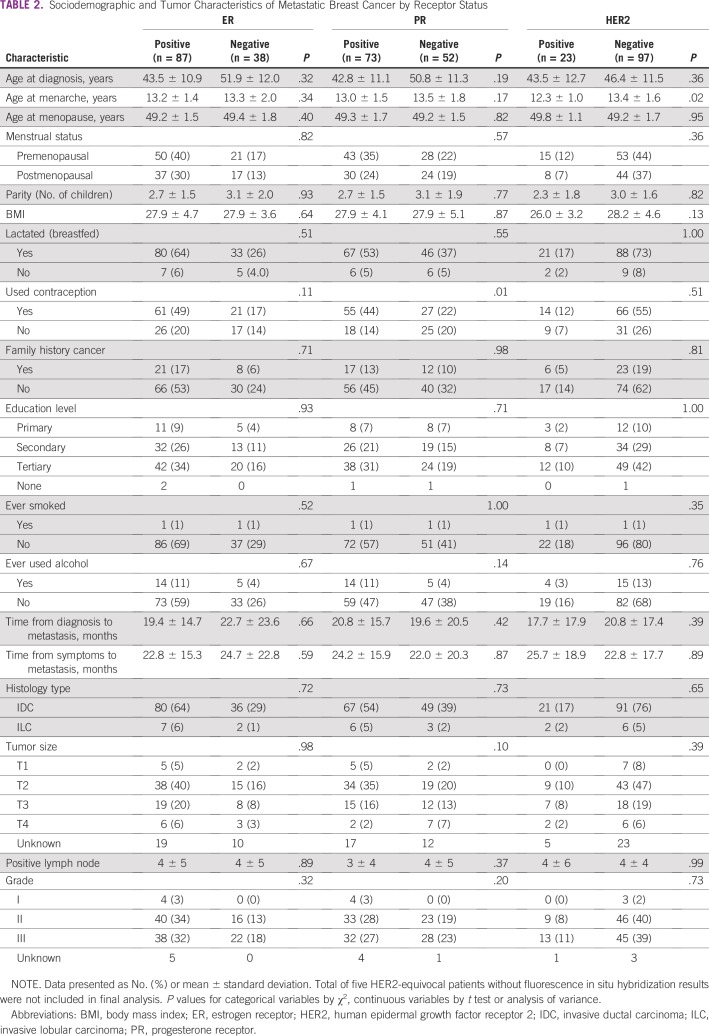
Sociodemographic and Tumor Characteristics of Metastatic Breast Cancer by Receptor Status

### Breast Cancer Metastatic Site

Metastasis to bone was associated with greater age at diagnosis (OR, 1.04; *P* = .02) and higher parity (OR, 1.24; *P* = .04), whereas metastasis to the brain was associated with early menopause (OR, 0.45; *P* = .04), lower parity (OR, 0.71; *P* = .04), and not breastfeeding (OR, 0.18; *P* = .01). However, concerning parity and age at menopause, they may not be clinically significant given the small differences in absolute values. Metastasis to the lung was associated with slightly longer time interval from initial diagnosis to metastatic disease (OR, 1.03; *P* = .05). The majority of patients with bone metastases had ER-positive and PR-positive cancers; however, these associations were nonsignificant. Brain metastases were inversely associated with ER-positive and PR-positive status (OR, 0.28; *P* = .01; and OR, 0.30; *P* = .02, respectively) and positively associated with triple-negative tumors (OR, 4.83; *P* = .01). Both brain and liver metastases were less common among patients with ER-positive and/or PR-positive, HER2-negative tumors (OR, 0.31; *P* = .02; and OR, 0.43; *P* = .03, respectively). However, liver metastases were found to be positively associated with ER-positive/HER2-positive tumors (OR, 8.02; *P* = .002) and HER2-positive status, irrespective of ER or PR status (OR, 2.75; *P* = .04; Tables 3 and 4).

**TABLE 3:**
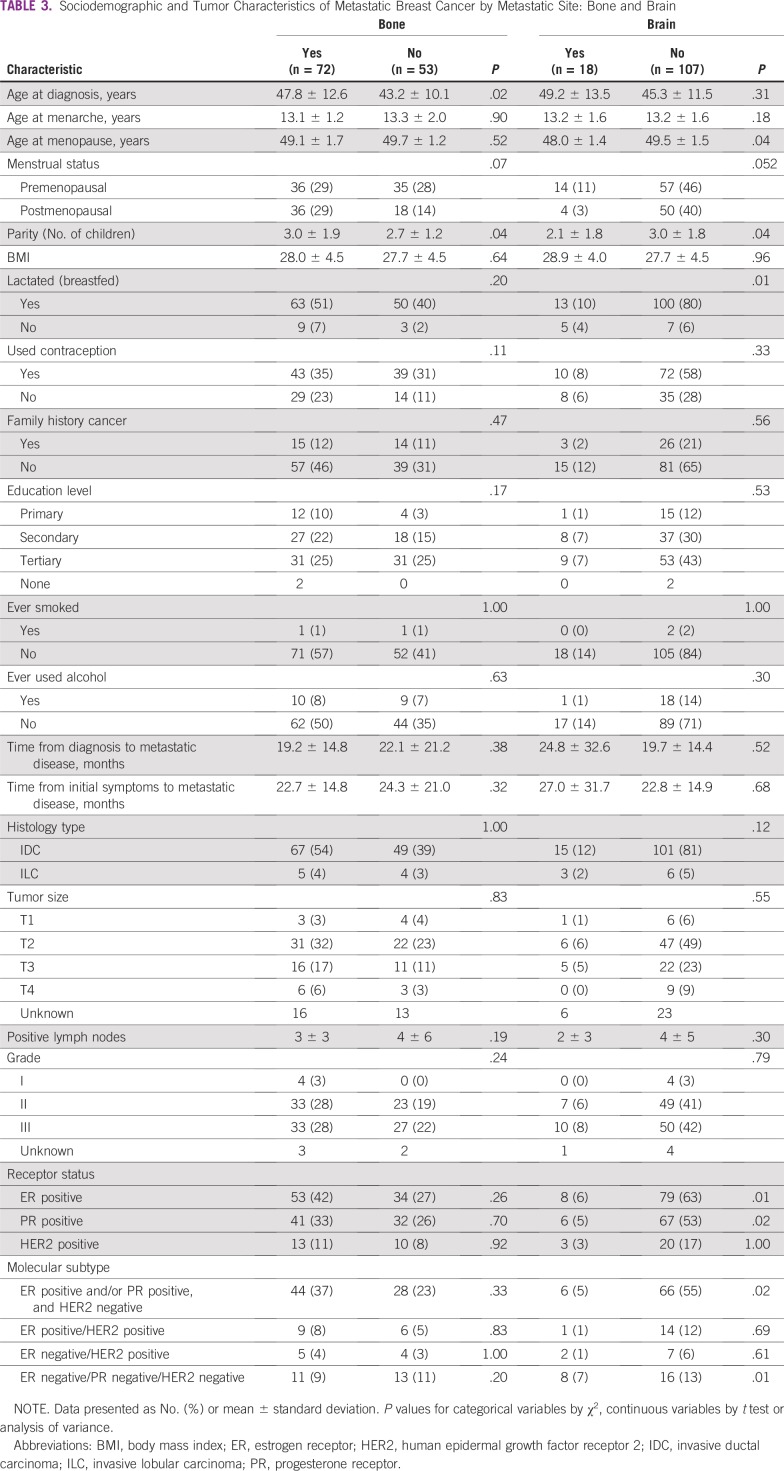
Sociodemographic and Tumor Characteristics of Metastatic Breast Cancer by Metastatic Site: Bone and Brain

**TABLE 4:**
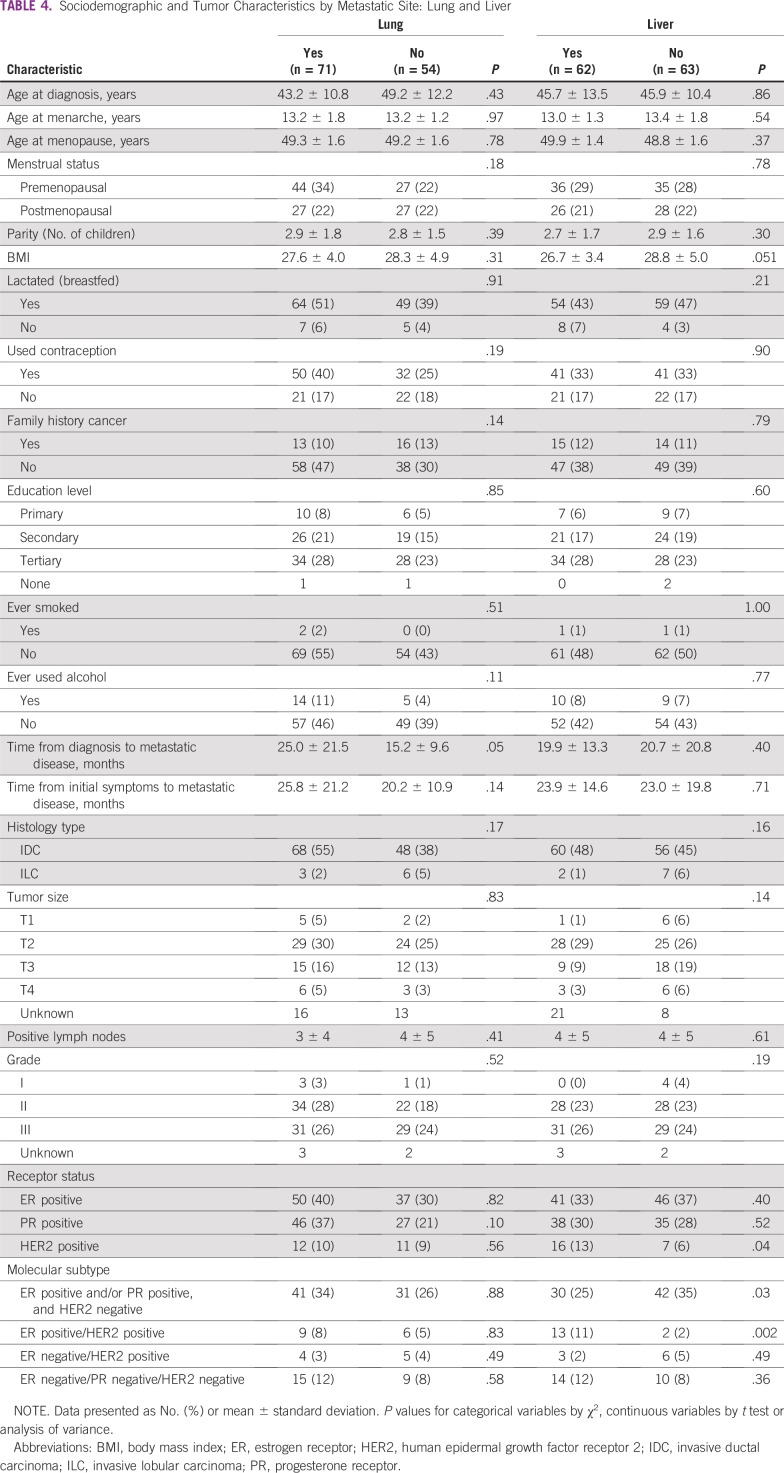
Sociodemographic and Tumor Characteristics by Metastatic Site: Lung and Liver

## DISCUSSION

Our study may be first to describe the metastatic patterns of a series of Kenyan patients with breast cancer in relation to demographic and tumor characteristics. Breast cancer can progress to metastatic disease, or stage IV, regardless of initial stage at diagnosis. In our study, patients diagnosed in stage III developed metastases sooner than those diagnosed in stage II, and patients who developed lung metastases had a longer duration of time to metastatic disease than those who did not develop lung metastases (irrespective of metastasis to other sites). Although there are many factors associated with breast carcinoma, including age at diagnosis, tumor grade and size, lymph node involvement, and receptor status, it is not always predictable which tumors will metastasize and to which site.^14^ There is a dearth of published literature regarding initial diagnosis and progression to metastatic disease; however, among many factors associated with risk of recurrence for breast cancer, stage at presentation remains one of the most important.^20^ Our findings may be a reflection of the biology of the tumors. Moreover, tumor size, which is used to determine stage at initial diagnosis, was found to be significantly associated with initial stage at diagnosis, as expected.

Patients diagnosed at later stages and those with negative hormone and HER2 receptor status were more common among older patients at diagnosis; however, none of these associations was significant. In a South African study, HER2-enriched tumors were more common in younger patients than were triple-negative tumors.^21^ In that study, as in ours, ER and PR status were not strongly associated with age at diagnosis.^21^ In our study, patients who developed metastasis to the bone were significantly associated with older age at diagnosis. In a literature review of bone metastasis from breast cancer, Pulido et al^22^ report that some studies found bone metastases associated with either younger^23^ or older age^24^ at diagnosis, whereas others found no association at all.^25^

Hormonal factors are known to play a role in breast cancer development.^26^ In our study, PR-positive breast cancer was associated with contraceptive use (OR, 2.83). Some studies have suggested that oral contraceptive use may increase breast cancer risk^26^; however, Westhoff et al^27^ report that long-term use of hormonal contraceptives has not been found to be associated with overall cancer risk. In our study, HER2-positive status was found to be associated with early menarche (OR, 0.65). In contrast, a 2003 study in the United Kingdom found HER2-positive status to be associated with older age at menarche.^28^ HER2-positive status is believed to be independent of hormonal risk factors, including early menarche, late menopause, and nulliparity.^28^ In addition, our study found that metastasis to bone was significantly associated with higher parity and that metastasis to the brain was significantly associated with early menopause, lower parity, and not breastfeeding. However, the small differences in absolute values for parity and age at menopause indicate the results may not be clinically significant—possibly related to the small sample size in our study. We have found no other study that has addressed the relationship between metastatic site and hormonal risk factors of breast cancer.

The receptor status and molecular subtype distribution in this series of Kenyan patients with metastatic breast cancer was similar to that in other studies of patients with breast cancer (with or without metastases). In a South African sample, most breast cancers were hormone receptor-positive, 54% were ER-positive and/or PR-positive, and HER2-negative tumors; 15% were ER-positive and/or PR-positive, and HER2-positive tumors; 11% were ER-negative, PR-negative, and HER2-positive tumors; and 20% were triple-negative tumors.^21^ Similarly, among our patients, 60% of breast cancers were ER-positive and/or PR-positive, and HER2-negative tumors; 12.5% were ER-positive/HER2-positive tumors; 7.5% ER-negative/HER2-positive tumors; and 20% were triple negative. Studies have suggested that breast cancer tends to be more aggressive and that its molecular subtype is more often triple negative in African populations than in Western populations.^5^ However, molecular subtypes vary widely across African populations, and receptor status in sub-Saharan African is overall similar to that in the West.^5,29^ In the South African study, late-stage and higher-grade tumors were more likely associated with triple-negative and ER-negative/PR-negative/HER2-positive tumors than with ER-positive and/or PR-positive, HER2-negative tumors.^21^ Triple-negative and HER2-positive tumors are considered more aggressive than those with positive hormonal receptors.^21^ In our sample, however, receptor status was not associated with either stage at diagnosis or tumor grade.

A number of studies have looked at the relationship between receptor status and metastatic site.^8,14-16^ Most studies reported that bone was the most common site of breast cancer metastasis. They also found that bone metastases were associated with ER-positive and/or PR-positive, HER2-negative and with ER-positive/HER2-positive tumors; that HER2-positive tumors were strongly associated with liver metastases; and that triple-negative tumors were associated with brain metastasis.^15,16^ Furthermore, brain metastases were more frequent in ER-negative/PR-negative/HER2-positive tumors than in ER-positive/PR-positive tumors.^14^ In our study, bone and lung metastases were not found to be significantly associated with either receptor status or molecular subtype, although we found significant associations between liver metastases and HER2-positive status, between liver metastases and ER-positive/HER2-positive tumors, and between brain metastases and triple-negative tumors. The relationship between distant metastatic site and molecular subtype of breast cancer may be due to homing factors that target a particular subtype to a specific organ or due to other undiscovered factors.^14^

There are a number of limitations in our study, including small sample size and retrospective study design. All of our sociodemographic information relied on chart review. In addition, because not all patients with breast cancer underwent surgery at the time of initial diagnosis, some data on tumor size, grade, and lymph node positivity were missing. Furthermore, there were 10 HER2-equivocal patients, five of whom did not have fluorescence in situ hybridization results. Those five were not included in the final HER2 analysis. Finally, 74% of our patients had metastases to multiple sites, and 26% had involvement of a single organ. Medical charts reviewed did not differentiate oligo from multiple metastases for single-organ metastatic involvement, and breast cancer metastasis to soft tissues such as the skin were not reported and therefore not analyzed.

Our study reports sociodemographic, pathologic, and tumor characteristics of a series of African patients with metastatic breast cancer from a tertiary cancer center in Nairobi, Kenya. Receptor status and molecular subtype distributions in this series of patients with metastatic breast cancer, as well as patterns of metastasis to certain sites in the body, were overall similar to what has been previously published. However, some findings concerning hormonal risk factors of breast cancer and metastatic site are unique and need additional exploration in larger patient populations.
